# Anaesthetic considerations in a child with rickets and craniosynostosis for linear strip craniectomy and frontal advancement

**DOI:** 10.4103/0019-5049.68394

**Published:** 2010

**Authors:** Rakesh Garg, Puneet Khanna, MP Pandia

**Affiliations:** Department of Neuroanaesthesiology, Neurosciences Centre, All India Institute of Medical Sciences, New Delhi, India

Sir,

Craniosynostosis is the result of premature closure of the cranial vault sutures.[1,2] Besides the functional impairment, there are often problems concerning the social integration of patients caused by the grotesque skull deformities.[3] Craniosynostosis can be nonsyndromic or syndromic (e.g. Apert’s syndrome and Crouzon’s syndrome).[2] Craniosynostosis has been observed in association with a number of maternal metabolic disorders including hyperthyroidism, rickets, Hurler syndrome, Morquio syndrome, beta-glucuronidase deficiency, mucolipidosis III, and a host of haematological disorders.[2–4] We report the anaesthetic considerations of a child of rickets and craniosynostosis planned for linear strip craniectomy and frontal advancement.

An 11-month-old male child weighing 6 kg presented in the neurosurgical clinics with chief complaints of progressively increasing bilateral proptosis since birth and abnormal shape of head since 3 months. He had a history of decreased sleep, increased sweating and increased frequency of micturition. Wrist X-ray showed widening of distal end of ulna, suggestive of rickets. He was administered vitamin D megadose. He continued to have persistently low calcium levels and phosphate levels.

The child was diagnosed with bilateral coronal craniosynostosis and it was planned to perform linear strip craniectomy and frontal advancement on the child. The respiratory and cardiovascular examination revealed no abnormality. On investigation, the haemoglobin was 7.2 g/dL and the child received two paediatric units of packed red cells transfusion and his haemoglobin improved to 11.2 g/dL. His serum calcium level was 8.9 mg/dL, phosphate level was 5.5 mg/dL and alkaline phosphate level was 980 IU.

In the operation room, routine monitors were attached. Anaesthesia was induced with 8% sevoflurane in oxygen and thereafter 22G intravenous access was secured. Then, 20 μg fentanyl and 20 mg rocuronium were administered intravenously. Airway was secured with size 4 mm ID endotracheal tube. Capnography and temperature monitoring were initiated Anaesthesia was maintained with 1% isoflurane in oxygen and nitrous oxide (50:50). The child was positioned for surgery. Top ups of fentanyl (total 30 μg) and rocuronium were administered, as guided by neuromuscular monitor. Intraoperatively, 50 mL of 20% mannitol was administered. Blood loss was 90 mL and was replaced with crystalloids adequately. The surgical duration was 4 hours. At the end of surgery, residual neuromuscular blockade was reversed and trachea extubated. The child had an uneventful recovery.

Our case of craniosynostosis was probably secondary to hypophosphataemic rickets. In our case, apart from cosmetic reason, considerations of the progressively increasing vision-threatening proptosis and risk of neurological impairment required urgent surgical repair.[1] The perioperative concerns in our patient included risk of corneal ulceration, airway related, rickets, air embolism, blood loss, and prolonged surgery with risk of head and neck oedema.

These patients should have had a complete multidisciplinary evaluation to rule out any syndromic association. Special attention should be directed to signs of increased intracranial pressure.[5] The poorly protected eye is exposed to the risk of corneal ulceration. The airway securing is difficult because of not only being an infant but also having abnormal facial features. The surgery on the head also had its implications for accidental extubation. The decreased level of calcium was a concern in view of neuromuscular function and risk of fractures during positioning. Venous air embolism has been a reported complication of craniosynostosis repair.[1,2] The inevitable blood loss is another concern in these procedures. Adequate venous access is critical. Embarrassment of venous drainage and lymphatics leads to significant oedema around the cranium in the first 24 hours. Generalised oedema can be minimised by optimising crystalloid and blood administration during the case.

The perioperative management of children who have these congenital malformations requires multidisciplinary care
